# Wide and high resolution tension measurement using FRET in embryo

**DOI:** 10.1038/srep28535

**Published:** 2016-06-23

**Authors:** Satoshi Yamashita, Takashi Tsuboi, Nanako Ishinabe, Tetsuya Kitaguchi, Tatsuo Michiue

**Affiliations:** 1 Department of Life Sciences (Biology), Graduate School of Arts and Sciences, The University of Tokyo, Tokyo, Japan; 2 Cell Signaling Group, WASEDA Bioscience Research Institute in Singapore (WABIOS), 11 Biopolis Way #05-02 Helios, Singapore 138667, Singapore; 3 Organization for University Research Initiatives, Waseda University, #304, Block 120-4, 513 Wasedatsurumaki-cho, Shinjuku-ku, Tokyo, 162-0041, Japan

## Abstract

During embryonic development, physical force plays an important role in morphogenesis and differentiation. Stretch sensitive fluorescence resonance energy transfer (FRET) has the potential to provide non-invasive tension measurements inside living tissue. In this study, we introduced a FRET-based actinin tension sensor into *Xenopus laevis* embryos and demonstrated that this sensor captures variation of tension across differentiating ectoderm. The actinin tension sensor, containing mCherry and EGFP connected by spider silk protein, was validated in human embryonic kidney (HEK) cells and embryos. It co-localized with actin filaments and changed FRET efficiencies in response to actin filament destruction, myosin deactivation, and osmotic perturbation. Time-lapse FRET analysis showed that the prospective neural ectoderm bears higher tension than the epidermal ectoderm during gastrulation and neurulation, and cells morphogenetic behavior correlated with the tension difference. These data confirmed that the sensor enables us to measure tension across tissues concurrently and with high resolution.

During early embryogenesis, dynamic morphogenesis such as invagination, bud formation, and tube closure push and pull cells, making and driven by cellular physical forces. These forces include tension anisotropy, tension or stiffness variance between cells, and pressure created by localized cell growth^1^,^2^,^3^,^4^,^5^. At the same time, cells sense tension, stiffness and other physical properties around them via adhesive and cytoskeletal structures^6^,^7^,^8^. These physical properties can influence tissue differentiation^9^,^10^. Now it is becoming clear that tension plays an important and interactive role in embryonic development^11^,^12^.

Several methods to measure intracellular or intercellular physical properties have been used. Micropipette aspiration and atomic force microscopy measure membrane tension or stiffness of a single cell. These techniques are not invasive, but can not distinguish the direction of the force. To measure the intercellular tension inside an embryo, laser ablation, hole punching^13^, and calculation from cellular geometry^14^ have been used. The hole punching method involves punching a hole in a cellular sheet and measuring the speed, width, and direction to which the hole spreads. The speed and width reflect tension on the cell sheet. Likewise, laser ablation is used to cut a cell-cell interface on a cellular sheet, followed by observation of how fast and wide the cut spreads. Tension measurement with the laser ablation and the calculation from cellular geometry showed a correlation between the tension on a cell-cell interface and the direction of the cell-cell interface. Therefore, these methods can be used to measure directional components of the cellular sheet tension; however, these methods are either invasive or their application is limited. A non-invasive measuring method using fluorescence resonance energy transfer (FRET)-based tension sensors has been developed in a cell culture system^15^,^16^,^17^,^18^. FRET-based tension sensors are made of a cell adhesion molecule with an inserted FRET domain, which contains two fluorescent domains connected by an elastic linker. The cell adhesion molecule can be vinculin^15^, actinin^16^, cadherin^17^,^18^, or *β*-spectrin^19^. When the sensor is under tension, the elastic linker is stretched and the fluorescent domains are separated, resulting in a decrease of FRET efficiency between the two fluorescent domains. These tension sensors can provide a subcellular resolution of measurement, but there are few reports using them in a tissue^19^,^20^, and development of an *in situ* functional tension sensor is needed.

In this study, we introduced a FRET-based tension sensor into *Xenopus* embryos and measured tissue tension. The sensor was first validated in cultured cells and embryos, and then applied to the gastrula/neurula ectoderm. The tension sensor demonstrated a clear distinction between prospective neural ectoderm and epidermal ectoderm. We also found that the neural and lateral epidermal ectoderms underwent similar morphogenesis, whereas their individual cells behaved differently.

## Results

### Validation of tension sensor in cultured cells and embryos

**Figure 1:**
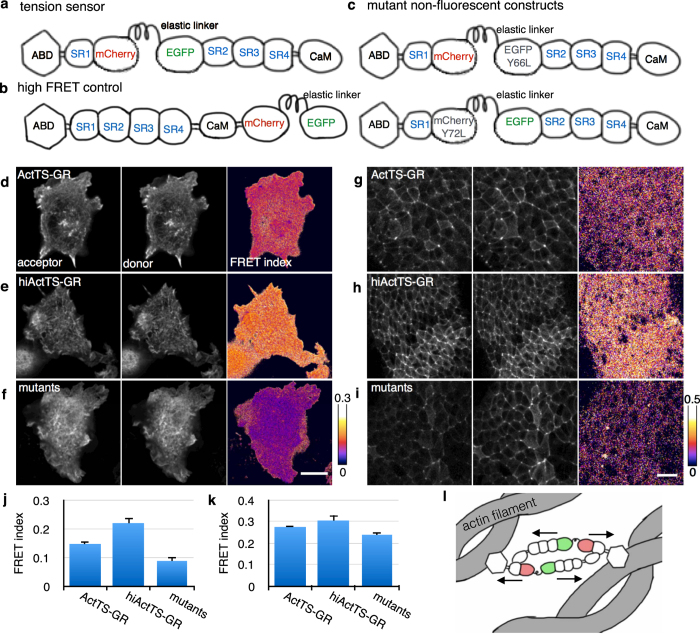
Actinin tension sensor in cultured cell and embryo. (**a**) Schematic diagram of tension sensor. The FRET domain was inserted between SR1 and SR2. ABD: actin binding domain. SR1-4: spectrin repeat domain. CLD: calmodulin-like domain. (**b**) High FRET control. The FRET domain was attached to the C-terminal of actinin with two linking amino acid residues. (**c**) Mutant non-fluorescent constructs. To break EGFP fluorescence, the 66^th^ tyrosine of the EGFP domain was replaced with leucine. To break mCherry fluorescence, the 72^nd^ tyrosine of the mCherry domain was replaced with leucine. Tyr66 of EGFP and Tyr72 of mCherry compose chromophores. (**d–i**) Acceptor images (left), donor images (center), and corrected FRET index images (right) of HEK cells (**d–f**) and *Xenopus* ectoderm (**g–i**) expressing ActTS-GR (**d,g**), hiActTS-GR (**e,h**), and a pair of ActTS-GR non-fluorescent mutants (**f,i**). (**j,k**) Quantification of the FRET index in HEK cells (**j**) and ectoderm (**k**). We measured >7 cells and >6 embryos per construct and values are average ± SD. (**l**) Schematic of the tension sensor in a cell. ActTS-GR makes an antiparallel dimer and bridge between actin filaments. Tension on actin filaments stretches FRET domain of ActTS-GR. Scale bars = 10 *μ*m in **f** and 50 *μ*m in **i**.

The first reported actinin tension sensor^16^ contained a FRET domain composed of Venus and Cerulean connected by an elastic linker derived from a spectrin repeat. This FRET domain was inserted between spectrin repeat domains 1 and 2 in *α*-actinin. However, this FRET domain had unexpectedly low FRET efficiency when the FRET domain alone was expressed in a cultured cell^16^. Therefore, we replaced the FRET domain with one containing mCherry and EGFP linked by an elastic linker derived from a spider silk protein^15^, and named it ActTS-GR (Fig. 1a). For a negative control that always shows high FRET efficiency, the FRET domain was attached to the C-terminal of an intact actinin (hiActTS-GR) (Fig. 1b). The tension sensor was validated in HEK cells and the dorsal ectoderm of *Xenopus* gastrula. HEK cells were transfected with plasmids encoding the sensor. Embryos were injected with 500 pg of the sensor mRNA into the dorsal animal marginal zone at the four-cell stage, and incubated until the gastrula stage. The tension sensor showed partial colocalization with actin filaments (Supplementary Fig. 1a,b). In the dorsal ectoderm, actin filaments were abundantly distributed around the apical-lateral adherens junction. The tension sensor also colocalized with the actin filaments, marking the cell-cell interfaces. To test their dynamics, we measured fluorescence recovery after photobleaching (FRAP). Recovery curves were identical between ActTS-GR and mCherry-tagged actinin in HEK cells and embryos (Supplementary Fig. 1c,d), indicating that ActTS-GR has similar dynamics to actinin.

Next, we calculated the FRET index of the tension sensors in living HEK cells and embryos. In addition to ActTS-GR and hiActTS-GR, we co-transfected a pair of mutant non-fluorescent ActTS-GR (Fig. 1c) into HEK cells (Fig. 1f) and co-injected them into embryos to measure inter-molecular FRET (Fig. 1i). As expected, hiActTS-GR had a high FRET index, the non-fluorescent mutants pair had a low FRET index, and ActTS-GR had an intermediate value both in HEK cells and embryos (Fig. 1d–k). In embryos, the FRET index of the mutants pair was exceptionally high, but we concluded that it was caused by autofluorescence of embryos since un-injected tissue also returned a FRET index similar to that of the mutants pair (data not show). To further confirm these results, we conducted acceptor photo-bleaching with fixed samples as an alternative method for estimating FRET efficiency without background noise^21^. After mCherry was bleached locally, the EGFP fluorescence intensity of ActTS-GR and hiActTS-GR increased in the mCherry bleached region and calculated FRET efficiencies were consistent with the FRET indices (Supplementary Fig. 2a,b,d–f,h). Importantly, FRET efficiency of the mutants pair was significantly lower in both HEK cells and embryos (Supplementary Fig. 2c,d,g,h), indicating that intermolecular FRET is low. These results suggest that ActTS-GR, when creating dimers and bridging actin filaments, was under tension, and the FRET domain was stretched (Fig. 1l).

**Figure 2:**
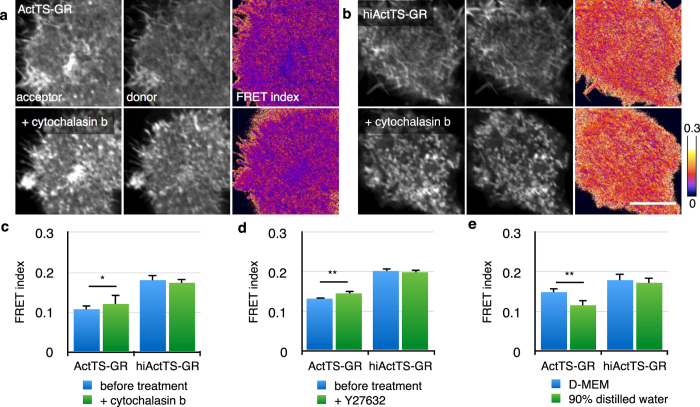
Tension measurement in HEK cells under experimental treatment. (**a,b**) Images of HEK cells expressing ActTS-GR (**a**) or hiActTS-GR (**b**) before cytochalasin was added (upper) and after incubation in 2.05 *μ*M cytochalasin b for 30 min (lower). Left: acceptor images. Center: donor images. Right: corrected FRET index images. Filament-like localization of the constructs was disrupted and they aggregated in speckles. (**c**) Quantification of the FRET index before and after cytochalasin was added. Values are average ± SD, n > 8. (**d**) Quantification of FRET index of cells expressing ActTS-GR or hiActTS-GR before Y27632 was added and after incubation in 20 *μ*M Y27632 for one hour. ROCK is an activator of myosin and Y27632 inhibits ROCK activity. Values are average ± SD, n = 12. (**e**) Quantification of FRET index of cells expressing ActTS-GR or hiActTS-GR under D-MEM or after incubation in 90% distilled water for 30 min. Values are average ± SD, n = 11. Scale bar = 10 *μ*m. *p < 0.05, **p < 0.0005.

We also examined the sensor function by experimentally changing the tension. HEK cells expressing either ActTS-GR or hiActTS-GR were examined with FRET before and after incubation in either the actin polymerization inhibitor, cytochalasin b, the Rho-associated protein kinase (ROCK) inhibitor, Y-27632, or a hypotonic solution. When the transfected cells were incubated in 2.05 *μ*M of cytochalasin b, the filament-like distribution disappeared and the fluorescence aggregated in clusters, indicating disruption of the actin filaments (Fig. 2a,b). The FRET index of ActTS-GR but not of hiActTS-GR was increased after the cells were treated with cytochalasin b (Fig. 2c). Likewise, treating HEK cells with 10 *μ*M of Y-27632 increased the FRET index of ActTS-GR, indicating a decrease in tension (Fig. 2d). In contrast, incubation in 90% distilled water decreased the FRET index of ActTS-GR but not of hiActTS-GR, indicating that the cells were swollen, and tension on the actin filament was increased (Fig. 2e). Together, these data indicated that ActTS-GR could respond to tension changes in HEK cells.

**Figure 3:**
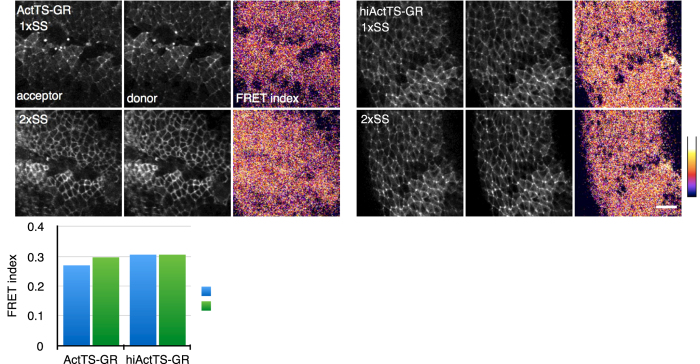
FRET efficiencies under different osmotic pressure. (**a,b**) Images of Xenopus ectoderm expressing ActTS-GR (**a**) or hiActTS-GR (**b**) in 1 × SS (upper) or 2 × SS (lower). Left: acceptor image. Center: donor image. Right: corrected FRET index image. (**c**) Quantification of FRET index of ectoderm under different osmotic pressures. Values are average ± SD, n > 4. Scale bar = 50 *μ*m. *p < 0.05.

In embryos, FRET images were obtained under a hypertonic solution. Switching the embryos from 1x Steinberg’s solution (SS) to 2x SS increased the FRET index of ActTS-GR but not of hiActTS-GR (Fig. 3), suggesting that the embryos were shrunk by the hypertonic challenge, releasing tension on the actin filament network across the embryonic surface. In case the constructs were toxic, injected embryos were incubated until the larval stage, and no apparent phenotype was observed (data not shown). These results confirmed that the construct functioned as a tension sensor inside cultured cells as in previously reported constructs, and also inside *Xenopus* embryos without evident toxicity.

### Tension measurement in *Xenopus* early embryo ectoderm

**Figure 4:**
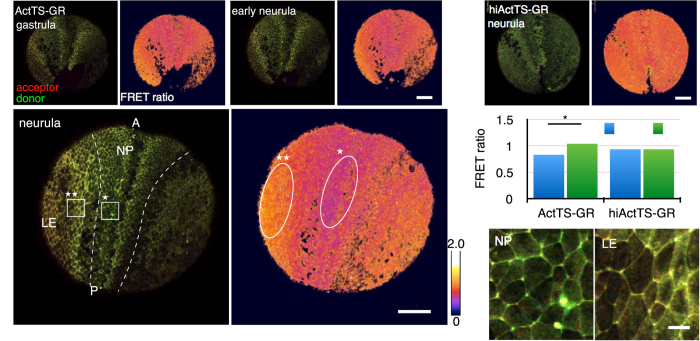
Tension on ectoderm and cells behavior during morphogenesis. (**a**) Time-lapse images of an embryo expressing ActTS-GR at gastrula, early neurula, and neurula stage (left), and its FRET ratio images (right). Vegetal view. Dashed line delineates the closing posterior neural plate (NP) and the lateral epidermis (LE). A: anterior. P: posterior. (**b**) Image of an embryo expressing hiActTS-GR at neurula stage (left) and its FRET ratio image (right). (**c**) Quantification of the FRET ratio in NP (oval with * in **a**) and LE (oval with ** in **a**) at the neurula stage. Values are average ± SD, n > 5. (**d**) Amplified image of NP (rectangle with * in **a**), and LE (rectangle with ** in **a**). Scale bars = 200 *μ*m in **a, b**, 20 *μ*m in **d**. *p < 0.05.

Having confirmed the functionality of the sensor, we then measured the variation in tension between differentiating tissues. In *Xenopus* gastrula, the dorsal ectoderm extends along the anterior-posterior axis and converges along the medial-lateral axis. The dorsal ectoderm becomes a neural plate in the neurula stage and undergoes neural tube closure, disconnecting from the epidermal ectoderm. At the same time, the vegetal ectoderm covers the whole embryo and forms the epidermis. The tensile properties of the neural plate and the epidermal ectoderm are different in an axolotl embryo^22^. Embryos were injected with 1 ng of ActTS-GR or hiActTS-GR mRNA into the animal marginal zone at the four-cell stage and observed from the gastrula stage to the neurula stage. Confocal images were taken from the vegetal view, and the tension was measured around a blastopore and in the dorsal marginal zone. We calculated the FRET ratio by dividing the acceptor fluorescence intensity by the donor fluorescence intensity^16^. This method cannot be used to evaluate FRET between the co-transfected/co-injected fluorescence mutants pair, but the acquisition of microscopic images is easy, and thus it is useful to analyze a large living tissue with high temporal resolution. We confirmed that FRET ratios calculated in the experiments described above were consistent with the FRET indices calculated in the same experiments (data not shown). The FRET ratio of ActTS-GR showed a clear separation between the prospective neural ectoderm and the epidermal ectoderm. The prospective neural ectoderm had a lower FRET ratio, and this difference was observed from the late gastrula stage (Fig. 4a–c).

**Figure 5:**
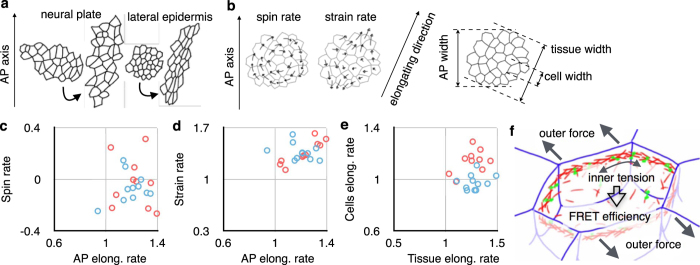
Deformation of cells and tissue in ectoderm. (**a**) Groups of cells in neural plate and lateral epidermis, at middle gastrula stage and early neurula stage. Cell membrane was tagged by membrane-tethered-GFP and the cells were traced. (**b**) Illustration of measured motion of cells and group of cells. Cells were tracked and it was decomposed into rotation (spin rate) and deformation (contraction and elongation, strain rate). The measured deformation gave direction of the elongation. For the cells and the groups of cells, widths along antero-posterior (AP) axis and elongating direction were measured to get AP elongation rate, cell elongation rate, and tissue elongation rate. (**c**) AP elongation rate versus antisymmetric spin rate. Blue plots: neural ectoderm. Red plots: epidermal ectoderm. (**d**) AP elongation rate versus symmetric strain rate, colored as **c**. (**e**) Tissue elongation rate versus cells elongation rate, colored as **c**. (**f**) Schematic diagram of ectodermal cell during morphogenesis. The cell is pulled by outer force (grey arrows outside the cell) and generating inner tension (grey arrow inside the cell). FRET efficiency of ActTS-GR indicates the inner tension. Cellular deformation depends on the outer force minus the inner tension.

In the neurula stage, the cell shapes of the epidermis and the neural plate were different. The cells in the neural plate remained isotropic in appearance, whereas the cells in the lateral epidermis were elongated along the antero-posterior axis (Fig. 4d). To investigate when and how this difference emerged, we injected 400 pg of membrane-tethered-GFP mRNA into an animal marginal zone at the four-cell stage and measured the deformation of cells and tissues (Fig. 5). We first traced cells manually and calculated tissue strain rates^23^. During the late gastrula stage, groups of cells in both prospective neural ectoderm and epidermal ectoderm underwent a similar rotation and deformation (Fig. 5c,d). They elongated roughly along the antero-posterior axis. Next, we measured the width of the cells and the groups of cells along the elongating axis (Fig. 5e). Both of them increased width in the epidermal ectoderm. In contrast, the width of the cells in prospective neural ectoderm did not change during tissue morphogenesis. Although the two differentiating tissues deformed similarly, their cells behaved differently. At gastrulation, the dorsal ectoderm is pulled antero-posteriorly by the underlying mesoderm^24^,^25^. A previous study using axolotl embryo measured the tensile property of the ectoderm by stretching the specimen of the ectoderm, and found that the epidermal ectoderm was easier to deform than the neural ectoderm^22^. Our data suggest that the cells in prospective neural ectoderm generated higher tension on actin filaments than the epidermal ectoderm cells, and the higher tension appeared as cell stiffness against outer forces (Fig. 5f). Both the prospective neural ectoderm and the lateral epidermal ectoderm were pulled antero-posteriorly by the mesoderm. The prospective neural ectoderm deformed by rearranging its stiff cells, whereas, in the epidermal ectoderm, the soft cells themselves deformed.

## Discussion

In summary, our experiments demonstrated that our tension sensor was sufficiently functional in embryos. ActTS-GR performed the best among FRET-based tension sensors we tested (data not shown). They were not invasive in that the injected cells engaged in normal tissue development and the embryos underwent the complete developmental process. The tension sensors distinguished between the prospective neural ectoderm and the epidermal ectoderm, when they were just beginning differentiation. The difference in measured tensions between the two tissues were consistent with the previous report^22^. Neural plate development has been reported to require a change in the cadherin complex components^26^,^27^, which might be responsible for the tension change.

In an epithelial tissue where actin filaments localized mainly around the apico-lateral adherens junction, ActTS-GR lined the cell-cell interfaces. With resolution high enough to distinguish each cell-cell interface, tension on the actin filament around the interface can be measured separately. Since the actomyosin network is a major generator of cortical contractile force^28^,^29^,^30^,^31^, the measured tension should correspond to the tension on the cell-cell interface. Similarly to laser ablation, correlation analysis between the direction of the cell-cell interface and its tensions will provide the directional components of the tension on a cell sheet. A recent study showed that medial myosin pulses also generate a force that is transmitted to the cell-cell interface^4^. The tension measurement now requires a subcellular and high temporal resolution. ActTS-GR measures inner tension. Measurement of the outer force should also be possible with sensors derived from cadherin, talin, or other cell-cell adhesion proteins. These FRET-based tension sensors have limitations. First, the tension range that can be measured by the sensor is narrow, and a variety of elastic linkers are needed to visualize tension distribution across other tissues. Second, it is difficult to express the tension sensor evenly across a large tissue by injecting mRNA, and establishment of a constitutively expressing transgenic strain is needed for accurate analysis.

This FRET-based sensor measures tension across tissue concurrently and with cellular resolution. The measurement can be performed in a living tissue. And the most importantly, this method does not require a special instrument, making it instantly available to all researchers. In future applications, this sensor will provide an easy but high spacial and time resolution sequential measurement of tension during morphogenesis, cell rearrangement, and differentiation.

## Methods

### Constructs

The DNA fragment of flagelliform sequence was synthesized, and cloned into the vector carrying CMV promoter. To generate a tension sensor module, mCherry was fused to N-terminus of flagelliform linker (GPGGA)_8_ and EGFP was fused to C-terminus of the linker^15^. The constructed module was fused between spectrin repeat domains of human alpha actinin 1^16^ and named ActTS-GR. For high FRET control, the module was fused with C-terminus and named hiActTS-GR. Non-fluorescent control (intermolecular FRET control) was generated by replacing an amino acid residue in chromophore of fluorescence domain. 66^th^ tyrosine of EGFP domain was replaced leucine and 72^nd^ tyrosine of mCherry domain was replaced to leucine, respectively. For mCherry-tagged actinin, mCherry was fused to C-terminus of human alpha actinin 1 with two linking amino acids between them. For mRNA *in vitro* synthesis, the constructs were cloned into the vector carrying SP6 promoter.

### Cell culture and transfection

HEK 293 cells were plated in 4-well coverglass chamber (Iwaki) in a total volume of 1 ml high-glucose DMEM culture medium, containing 10% heat-inactivated FBS, 100 U/ml penicilin and 100 *μ*g/ml streptomycin, and incubated at 37 °C and 5% CO_2_. For transfection, 1:1 mixture of 0.323 mg/ml polyethylenimine (Wako) and 100 ng/*μ*l DNA with 75 mM NaCl was incubated for 30 min at room temperature, and 25 *μ*l of the mixture was added into 0.5 ml cell culture. Cells were incubated for 1–2 days after transfection.

### *Xenopus* embryos and micro-injection

mRNA were injected into 4 sites of dorsal animal marginal zone or 8 sites of animal marginal zone. Injections were performed in a 5% ficoll solution. Embryos were cultured in 1x Steinberg’s solution. For osmotic perturbation, embryos were transferred between dishes with different concentration of SS in them.

### Immunostaining

For actin filaments staining, HEK cells expressing ActTS-GR were incubated with 1 *μ*M Sir-actin (SPIROCHROME) for 30 minutes at room temperature. For embryos, vitelline membrane were removed surgically and the embryos were immediately fixed in MEMFA for 30 minutes. The samples were permeabilized in 1% PBT for an hour, followed by an incubation in blocking buffer for an hour. The samples then stained with Alexa Fluor 488 phalloidin at 4 °C overnight, washed with PBS.

### Confocal imaging

An Olympus FV1200-D confocal microscope was used for imaging all samples in this study. An UPLSAPO40XS silicon immersion objective was used for imaging HEK cells. An UPLSAPO10X2 objective was used for imaging embryos. Embryos were imaged on 35 mm/glass base dish (Iwaki), in Steingerg’s solution for FRET imaging and immuno-stained samples, or embedded in 1.2% UltraPure L.M.P Agarose (invitrogen)/Steingerg’s solution for membrane-tethered-GFP injected embryos. Immuno-stained images of HEK cells show maximum intensity projections of Z-stack covering lower part of the cells. Images of HEK cells for FRET analysis were single slice. Images of embryos show maximum intensity projections of Z-stacks.

### FRAP analysis

The initial 4 images were acquired and then a spot in a cell (HEK cells) or a spot on a cell-cell junction (embryos) was photo-bleached for mCherry. The samples were then imaged every 0.128898 seconds for 12.11641 seconds (HEK cells) or every 0.064904 seconds for 6.10097 seconds (embryos). Average intensity in the spot was measured by FV10-ASW, the microscope controller software.

### FRET analysis

FRET index and FRET ratio are the quantitative indicator of the FRET efficiency. FRET index was calculated with a custom script of ImageJ. The calculation algorithm follows the intensity-based FRET index calculation^32^ with an assumption that unquenched donor (*D*) is proportional to acceptor (*A*) signal intensity. Acceptor and donor spectral bleed-through (ASBT and DSBT) were obtained from *A* and quenched donor q*D* as ASBT = *c*_*a*_*A* and DSBT = *c*_*d*_q*D*, where coefficients *c*_*a*_ and *c*_*d*_ were obtained from images of cells and embryos expressing non-fluorescent mutant constructs. Then pure FRET (PFRET) is PFRET = uFRET−ASBT−DSBT, where uFRET is uncorrected FRET (an image of the acceptor fluorescence channel excited by laser for the donor). Then *D* is *D* = Q*d* + PFRET × *γ*, where *γ* is a factor of the fluorescent pair, and the FRET efficiency *E* is proportional to FRET index:


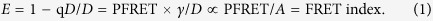


For an embryo, FRET calculation was performed on every single slice and the maximum FRET index of Z-stack was projected.

FRET ratio was calculated on ImageJ, dividing *A* by q*D*. Prior to dividing the acceptor image by the donor image, an area that did not express tension sensors was masked, where the mask was derived from the donor image to cover an area under the lower threshold. The lower threshold was determined based on an intensity of marginal zone. The calculation was done with images of the maximum intensity projection of Z-stacks. When the tension is low and the two fluorescent domains are enough close to each other, excited EGFP lose an energy by FRET so that the fluorescence intensity of EGFP was lowered. When the tension is high, FRET does not occur and EGFP emits full fluorescence. Therefore, ratio between EGFP and mCherry reflects the FRET efficiency. Statistical significance was assessed by student t-test on Numbers (Apple).

### Acceptor photo-bleaching

For acceptor photo-bleaching, HEK cells were fixed in 4% paraformaldehyde/PBS for 20 minutes and then washed with PBS. Embryos were fixed in MEMFA for 1 hr at room temperature and then washed in PBS. FRET efficiency was calculated based on increase of donor fluorescence that was quenched before acceptor was bleached^21^.

### Calculation of tissue and cell deformation

For each cell, its center location in plane *x,y* was taken manually, and for its velocity (*v*_*x*_, *v*_*y*_), change of the location from 40 minutes before was taken as an average velocity and used for calculation below. A group of 20–30 cells were chosen so that the cells were adjacent to each other and the group did not split in two groups along the cell rearrangement. A tissue velocity gradient tensor,


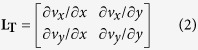


was derived from the locations and velocities of the cells, where the partial derivative of *v*_*x*_ or *v*_*y*_ respect to *x* or *y* was given by a coefficient of a line fitting to the distribution of them, i.e., 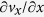 was given by a trend line fitting to the data projected on a plane *x*, *v*_*x*_. **L**_**T**_ was then decomposed into an antisymmetric spin **Ω**_**T**_ 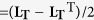, and a symmetric strain rate tensor 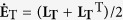. An elongation rate and its direction were given by an eigenvalue and eigenvector of 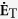. The eigenvalue 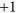 was taken as the symmetric strain rate value, and the (1,2) element of **Ω**_**T**_ was taken as the spin rate value. An antero-posterior, tissue, and cell elongation rate represented changes of bounding box width. The bounding boxes were tilted along the antero-posterior axis or the elongating direction. The antero-posterior axis were determined according to a closing neural tube. Tissue and cells elongating direction was corrected by **Ω**_**T**_. Since an embryo was spherical, the projection on a plane caused a distortion, but it could be ignored as long as relative elongation rates between cells and its group was issued.

## Additional Information

**How to cite this article**: Yamashita, S. *et al*. Wide and high resolution tension measurement using FRET in embryo. *Sci. Rep.* **6**, 28535; doi: 10.1038/srep28535 (2016).

## Supplementary Material

Supplementary Information
