# Supplementary material for: Wide and high resolution tension measurement using FRET in embryo
Source: Sci Rep. 2016 Jun 23;6:28535. doi: 10.1038/srep28535 (PMC4917836; doi:10.1038/srep28535)
Supplement: Supplementary Information [file srep28535-s1.pdf]

Supplementary Information

Wide and high resolution tension measurement using FRET in embryo

Satoshi Yamashita[1], Takashi Tsuboi[1], Nanako Ishinabe[1] , Tetsuya Kitaguchi[2,3] & Tatsuo Michiue[1]

[1]Department of Life Sciences (Biology), Graduate School of Arts and Sciences, the University of Tokyo, Tokyo, Japan.

[2]Cell Signaling Group, WASEDA Bioscience Research Institute in Singapore (WABIOS), 11 Biopolis Way #05-02 Helios, Singapore 138667, Singapore.

[3]Organization for University Research Initiatives, Waseda University, #304, Block 120-4, 513 Wasedatsurumaki-cho, Shinjuku-ku, Tokyo, 162-0041, Japan.

Table of contents:

1. Supplementary Figure 1 - Tension sensors localization and dynamics

2. Supplementary Figure 2 - Acceptor photo-bleaching

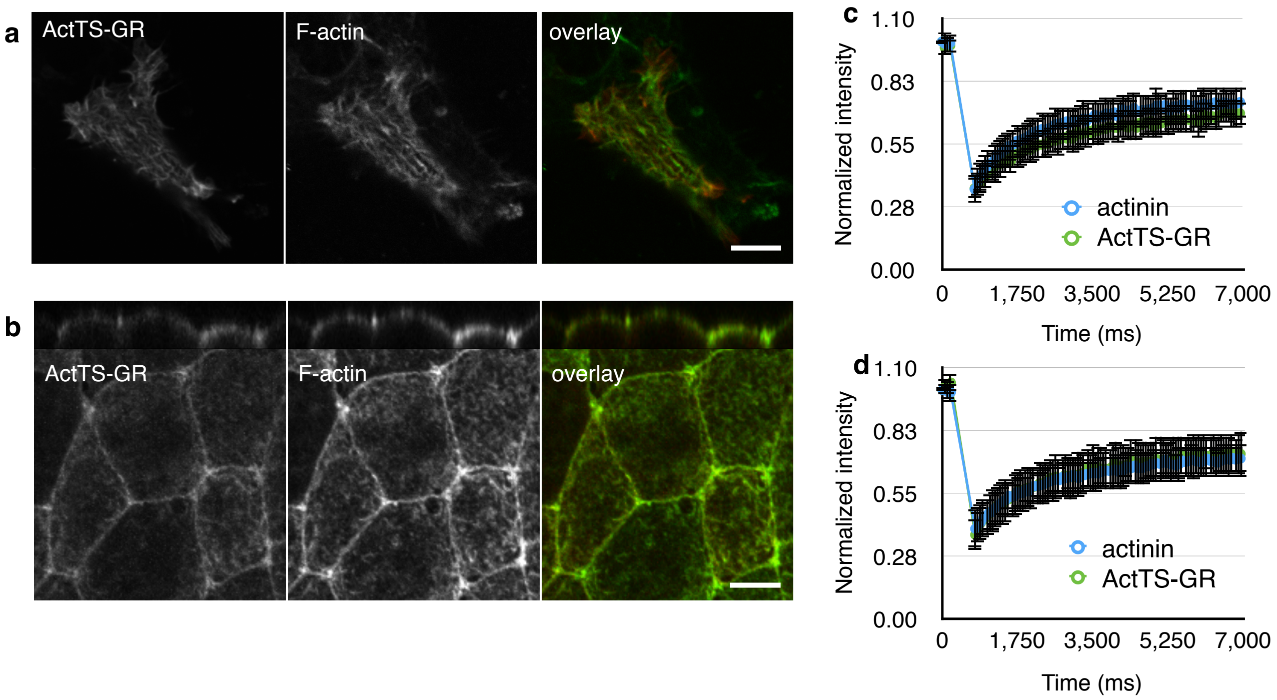

Supplementary Figure 1

Tension sensors localization and dynamics.

(**a, b**) Localization of ActTS-GR in HEK cell (**a**) and in *Xenopus* ectoderm (**b**). Upper panels of **b** show side view projection of a 3D image stack. Left: ActTS-GR. Center: actin filaments. Right: overlay. The tension sensors co-localized with the actin filaments. (**c**) HEK cells were transfected with ActTS-GR or mCherry-tagged actinin, and a spot in a cell was photo-bleached for EGFP and fluorescence recovery was measured. ActTS-GR showed the same recovery curve with actinin, indicating that ActTS-GR was transported normally. (**d**) FRAP measurement in ectoderm expressing ActTS-GR or mCherry-tagged actinin. A spot on a cell-cell interface was photo-bleached. ActTS-GR showed normal dynamics. Values are average ± SD, n = 10. Scale bar = 10μm.

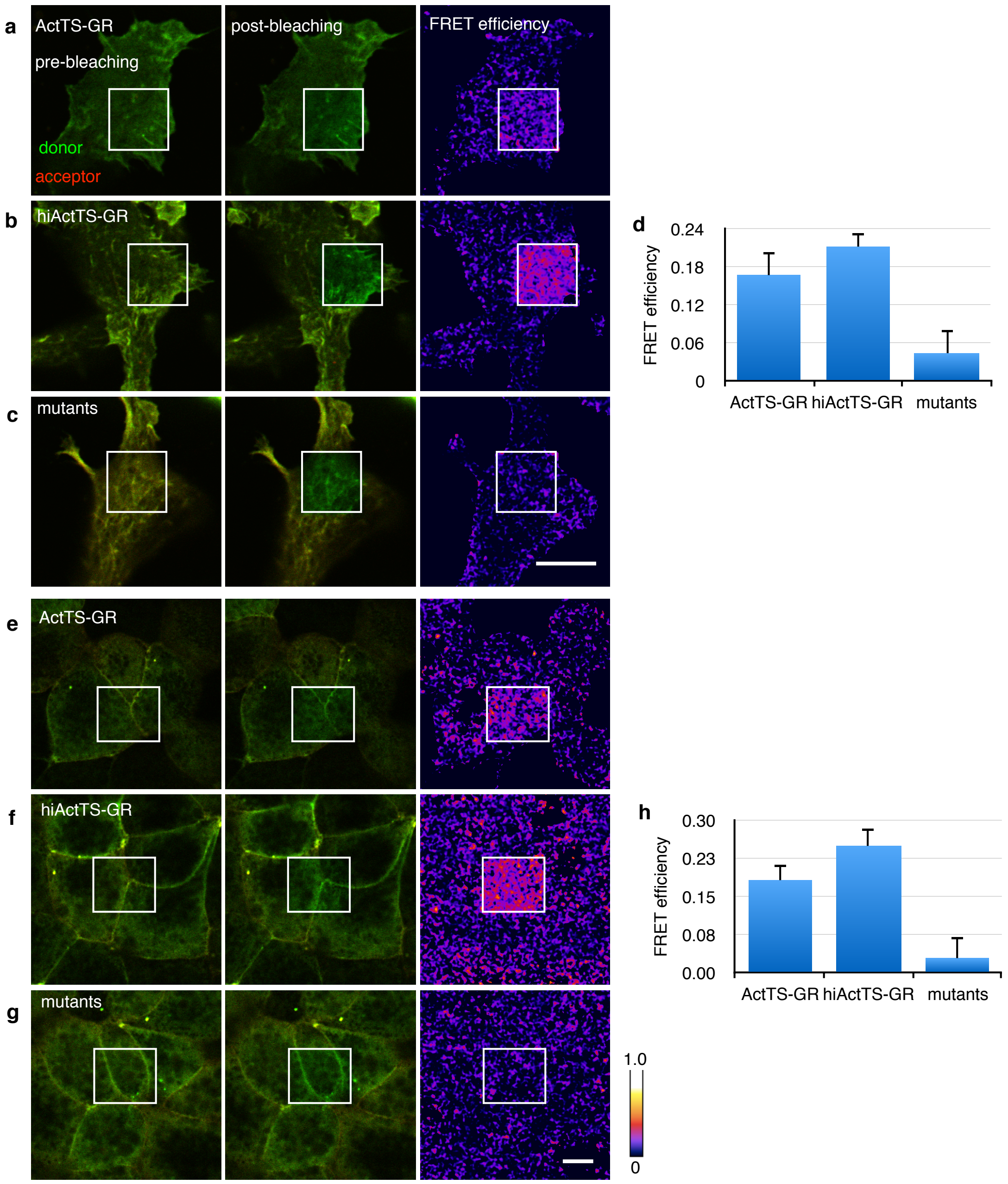

Supplementary Figure 2

Acceptor photo-bleaching.

(**a-c**) Images of HEK cell expressing ActTS-GR (**a**), hiActTS-GR (**b**), or pair of fluorescence mutants ActTS-GR before bleaching (left), after bleached (center), and their FRET efficiencies (right). mCherry in a white rectangle was bleached. (**d**) Quantification of FRET efficiency. Values are average ± SD, n = 10. (**e-g**) Images of *Xenopus* ectoderm expressing ActTS-GR (**e**), hiActTS-GR (**f**), or mutants pair (**g**) before bleaching (left), after bleaching (center), and their FRET efficiencies (right). (**h**) Quantification of fluorescence increase in ectoderm. n = 10. Scale bars = 10μm.
